# TSH Receptor Antibody Test Utilization Patterns From a National Reference Laboratory: TRAb, TSI, or Both?

**DOI:** 10.1210/clinem/dgaf330

**Published:** 2025-06-07

**Authors:** Heather A Nelson, Kelly Doyle, Joely A Straseski

**Affiliations:** Department of Pathology, University of Utah Health, Salt Lake City, UT 84108, USA; Department of Pathology, University of Utah Health, Salt Lake City, UT 84108, USA; Department of Pathology, University of Utah Health, Salt Lake City, UT 84108, USA

**Keywords:** thyroid-stimulating hormone receptor antibodies, TRAb, TSI, utilization, clinical guidelines

## Abstract

**Context:**

There are currently 2 classes of thyroid-stimulating hormone receptor (TSHR) antibody assays, namely TSHR antibody (TRAb) assays and thyroid-stimulating immunoglobulin (TSI) assays. Clinical guidelines do not currently specify appropriate use of TSHR autoantibody tests in the diagnosis of hyperthyroidism, which may result in paired orders for both tests with the possibility of discordant results and excessive costs.

**Objective:**

This work aimed to evaluate the clinical and analytical redundancy of paired TSHR autoantibody testing—specifically TRAb and TSI assays—in the diagnosis of autoimmune-mediated hyperthyroidism, by analyzing the frequency of paired orders, assay concordance, and clinical correlation across a large cohort of patient encounters.

**Methods:**

Over 189 000 patient encounters with TRAb and TSI bioassay (TSI-BA) or TSI bridge immunoassay (TSI-Br) test orders were examined to assess the frequency of paired orders and qualitative agreement of TRAB/TSI. A chart review was performed on a subset of patients for clinical correlation. Lastly, a cost analysis was performed to estimate the financial burden of unnecessary paired testing.

**Results:**

TRAb and TSI were co-ordered on the same encounter in 14.3% of TRAb/TSI-BA orders and 17.4% of TRAb/TSI-Br orders. Qualitative comparison showed discordance in 12.5% (1590) of TRAb and TSI-BA paired orders and 6.6% (1149) of TRAb and TSI-Br paired orders. Based on patient free thyroxine and TSH concentrations and disease status, the TSI assays aligned better with hyperthyroidism and confirmed Graves disease diagnoses. Paired orders resulted in a 31% to 325% increase in potentially unnecessary testing costs.

**Conclusion:**

We observed good clinical and analytical agreement between TRAb and TSI assays, suggesting that paired orders for TRAb and TSI are redundant in the assessment of autoimmune-mediated hyperthyroidism. Use of a single test to assess TRAb would be appropriate in most scenarios and may lead to considerable savings of health care dollars.

With continuously increasing costs, health care organizations are challenged to provide high-quality care while minimizing expenses. Improving laboratory test utilization is 1 approach to meet these challenges (1). Inappropriate utilization may include excessive or redundant test ordering or orders that are contrary to clinical guidelines published by regulatory or professional societies (2-4). Referencing clinical practice guidelines to direct appropriate testing is often limited by vague or conflicting recommendations, a lack of adequate evidence-based methods, or the absence of testing recommendations altogether (5). Notably, testing innovation and clinical implementation of new tests outpace guideline revisions (1).

Current American Thyroid Association (ATA) guidelines for diagnosis and management of hyperthyroidism recommend testing for thyroid-stimulating hormone receptor (TSHR) antibodies (TRAbs) to help determine disease etiology (6). In those with Graves disease (GD), the guidelines suggest this testing may be used to monitor disease activity, guide treatment, and provide prognostic insight into disease severity and risk of relapse (6, 7). This guideline, however, does not indicate which test(s) should be used to measure TRAb.

TRAbs are IgG immunoglobulins that group into 2 categories: (1) thyroid-stimulating immunoglobulins (TSIs), which bind to the TSHR and mimic the biological action of TSH, including stimulating production of thyroid hormones, and (2) blocking TRAbs that bind to and block the TSHR, resulting in absence of thyroid hormone production (8).

Just as there are 2 different categories of TRAbs, there are 2 main methods used to measure their presence: (1) anti-TSHR assays, generally called “TRAb” assays, which indiscriminately measure TSHR autoantibodies (stimulating or nonstimulating) and (2) “TSI” assays, which specifically measure stimulating autoantibodies. Third-generation TRAb assays are immunoassays that utilize a labeled human TRAb clone to compete with TRAbs in the patient sample for binding to immobilized TSHR (9). Therefore, this assay may also be referred to as a thyroid binding–inhibiting immunoglobulin assay; however, it does not distinguish between stimulating and nonstimulating antibodies.

While TRAbs are traditionally measured using immunoassays on automated, high-throughput platforms, TSI may be measured using 1 of 2 methods: (1) cell-based TSI bioassays (TSI-BAs) or (2) a bridging TSI immunoassay (TSI-Br). The TSI-BA uses cultured cells expressing human TSHR fused to a G-coupled protein receptor. Stimulation of the TSHR by TSI in the patient sample results in generation of cyclic adenosine monophosphate (cAMP) in cultured cells, serving as a direct measure of the net stimulating activity of TRAbs in the patient specimen (10, 11). In contrast, the TSI-Br employs 2 TSHR chimeras harboring binding sites specific to stimulating immunoglobulins, which sandwich stimulating TRAbs in the patient specimen. Modification of the membrane-proximal part of the TSHR binding site in these receptor chimeras precludes binding of nonstimulating or inhibiting TRAbs, so that only binding of stimulating antibodies is detected (12). A key difference in the 2 TSI assay formats is that the cell-based bioassays measure net stimulatory activity on the TSHR, whereas the TSI-Br measures the total concentration of stimulating antibodies, based on antibody binding to receptor chimeras, independent of the activity of nonstimulating antibodies that may be present.

It is unclear if there is any diagnostic advantage to testing a patient for TRAb vs TSI, or if measuring both is beneficial. Several reports have shown good overall agreement and diagnostic performance between TRAb and TSI (TRAb/TSI) assays (13-16), while others have shown TSI may outperform TRAb in patients undergoing treatment for GD or those with Graves orbitopathy (16, 17). The prevailing data suggest 1 TRAb test may be sufficient when evaluating a patient for GD. Given the lack of clarity in the clinical guidelines, we hypothesized there may be inappropriate test utilization and, more specifically, redundant ordering of TRAb/TSI assays. Therefore, the objective of this study was to evaluate ordering patterns of TRAb/TSI, specifically when both were ordered for the same patient during the same encounter. In paired orders, qualitative test results were compared to determine if there was clinical utility in ordering both tests compared to 1 test. Lastly, a cost analysis was performed to determine the economic impact of ordering both tests during the same encounter.

## Materials and Methods

### Data Analysis

Retrospective data review was performed on all orders for TRAb and/or TSI performed at a reference laboratory during 2 equivalent timeframes when either TSI-BA or TSI-Br was the predominant method used for TSI measurement in our laboratory. From these data sets, the frequency of ordering both TRAb/TSI during the same encounter was evaluated. Data were divided into 2 groups based on the availability of TSI assays in our laboratory: (1) orders for TRAb and TSI-BA (TRAb/TSA-BA) and (2) orders for TRAb and TSI-Br (TRAb/TSI-Br).

**Figure 1.**
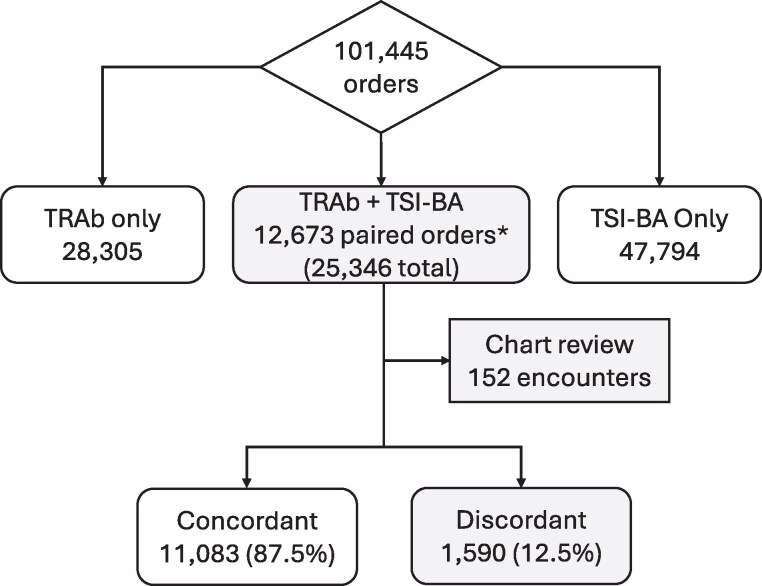
Flow diagram detailing the data extraction for orders of TRAb and TSI-BA. *Paired orders, where a “paired” order includes 1 order for TRAb and 1 order for TSI-BA on the same encounter (2 total orders per pair).

A total of 101 445 orders (78 947 unique patients) for TRAb and TSI-BA were received during the selected time. Of these orders, 28 305 were for TRAb only, 47 794 were for TSI-BA only, and there were 12 673 paired orders, with an order for both TRAb and the TSI-BA on the same encounter (2 total orders per pair) (Fig. 1). Patients ranged from 0 to 101 years of age (median 46 years) and were 77% female. In total, 590 patients in our data set had multiple (2-11) TRAb/TSI-BA paired orders submitted over the study period. No orders were excluded from the study.

**Figure 2.**
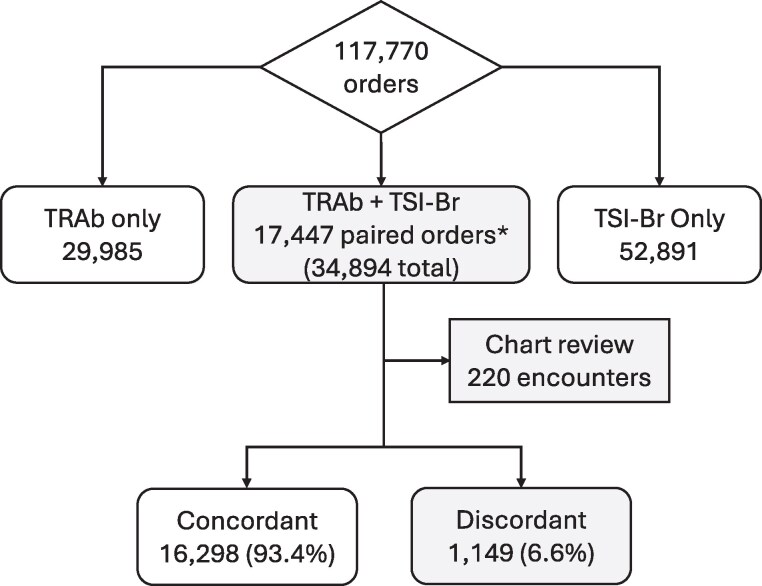
Flow diagram detailing the data extraction for orders of TRAb and TSI-Br. *Paired orders, where a “paired” order includes 1 order for TRAb and 1 order for TSI-Br on the same encounter (2 total orders per pair).

The TRAb and TSI-Br dataset included 117 770 orders (representing 87 487 unique patients). Of these orders, 29 985 were for TRAb only, 52 891 were for TSI-Br only, and 17 447 paired orders, with an order for both TRAb and the TSI-Br on the same encounter (2 total orders per pair) (Fig. 2). Patients ranged from 0 to 102 years of age (median 46 years) and were 79% female. A total of 953 patients in our data set had multiple (2-9) TRAb/TSI-Br paired orders submitted over the study period. No orders were excluded from the study.

For all patients with orders for both TRAb/TSI, qualitative agreement between test results was first assessed using manufacturer-defined cutoffs (see “Measurement of Analytes”). A TRAb/TSI result less than or equal to the cutoff was classified as “negative” while a result greater than the cutoff was classified as “positive.” A discordant result is defined as 1 positive TRAb or TSI test result paired with a negative result on the alternative antibody test on the same patient encounter. To better understand qualitatively discordant TRAb/TSI results at manufacturer cutoffs, a secondary analysis was performed to evaluate the proximity of each result to the cutoff used for each assay, realizing the higher likelihood of discordant results near the clinical cutoff. “Low positive” results were defined as greater than the manufacturer's cutoff but less than 3 times the upper limit of normal (ULN) (TRAb 1.76-5.24 IU/L; TSI-BA 123-365%; TSI-Br 0.55-1.61 IU/L), while results greater than or equal to 3 times the ULN were defined as “high positive” (TRAb ≥5.25 IU/L; TSI-BA ≥366%; TSI-Br ≥1.62 IU/L). Results were divided into negative, low positive, and high positive categories for comparison of TRAb and TSI results. The 3 times the ULN cutoff was chosen to enhance specificity based on national guidelines for management of thyroid disease in pregnant women that recommend increased fetal surveillance when TRAb levels exceed 2 to 3 times the ULN (18, 19).

### Clinical Correlation

These data sets were further evaluated to identify patients for whom chart review was available with free thyroxine (FT4) testing ordered in addition to TRAb/TSI during the same encounter. This represented 152 patients in the TRAb/TSI-BA group and 220 patients in the TRAb/TSI-Br group. From this subset, a chart review was performed on patients with discordant TRAb/TSI results to assess their TSH concentrations, thyroid-related diagnoses, and prescriptions for thyroid medication at the time of testing. For the test with a positive result, a medical diagnosis of GD was considered the primary indicator of “better” test performance in discordant cases. Elevated FT4 and suppressed TSH concentrations (when available) provided supportive evidence of “better” test performance. Data review was performed under approval by the University of Utah Institutional Review Board (IRB 001573160157316).

### Cost Analysis

To evaluate costs associated with ordering both TRAb/TSI, average list prices for each test were obtained in January 2024 from US-based reference laboratories that offer these methods (TRAb [n = 4], TSI-BA [n = 2], TSI-Br [n = 2]). Pricing did not represent any group or high-volume discounts. List prices were averaged and rounded to the nearest whole dollar.

### Measurement of Analytes

#### TSHR antibody

TRAb was measured using the Roche e601 Elecsys Anti-TSHR (TRAb) assay (Catalog # 08496609190, RRID:AB_3696448, Roche Diagnostics, Indianapolis, IN) according to the manufacturer's instructions (20). This is a competitive electrochemiluminescent immunoassay in which TRAb present in the patient specimen binds to an immobilized TSH receptor and inhibits binding of a ruthenium-labeled human thyroid-stimulating monoclonal autoantibody. A cutoff value of 1.75 IU/L or less indicated a negative result for TRAb (20). This cutoff suggested by the manufacturer had a reported sensitivity and specificity of 97% and 99%, respectively (established in 436 apparently healthy individuals, 210 individuals with non-Graves thyroid diseases, and 102 individuals with untreated GD (21)). There was no change in test methodology or cutoff over the duration of the study.

#### TSI bioassay

The TSI-BA method was described previously (11). This method uses a Chinese hamster ovary cell line expressing a recombinant form of the TSHR. Receptor stimulation by TSI initiates a signaling cascade and cAMP production, which is measured by immunoassay (DiscoverX Hit-Hunter cAMP XS, Catalog # 90-0041AR, RRID:AB_3696708) and compared with a reference sample. Results are expressed as a percentage of basal activity in the reference sample. A cutoff value of 122% or less indicated a negative result using this method with a sensitivity of 92.9% and specificity of 99.2% when comparing normal sera to Graves sera (11). There was no change in test methodology or cutoff over the duration of the study.

#### TSI bridge immunoassay

TSI-Br was performed using the Siemens Immulite 2000 XPi TSI assay (Siemens, Tarrytown, NY) according to the manufacturer's instructions (22). This assay utilizes 2 recombinant human TSHR constructs for the capture and detection of thyroid-stimulating autoantibodies in a bridging format. The capture TSHR chimera was modified to remove the major epitope for inhibitory TRAbs and immobilized on a polystyrene bead. The detector TSHR chimera is conjugated to alkaline phosphatase. In the presence of stimulating TRAb in the patient sample, a bridge is formed between the patient antibody and the 2 TSHR constructs, which is detected by the alkaline phosphatase tag. A cutoff value of 0.54 IU/L or less indicated a negative result using this method (22). This cutoff suggested by the manufacturer had a reported sensitivity and specificity of 98.6% and 98.5%, respectively (established in 361 individuals with untreated GD and 404 individuals with non-Graves thyroid disease or other autoimmune diseases (22)). There was no change in test methodology or cutoff over the duration of the study.

#### Free thyroxine

FT4 was measured using the Roche e601 Elecsys FT4 II assay (Catalog # 06437281160, RRID:AB_2924686, Roche Diagnostics, Indianapolis, IN) according to the manufacturer's instructions (23). This is a competitive electrochemiluminescent immunoassay in which FT4 present in the patient specimen competes with biotinylated T4 for binding to a ruthenium-labeled T4 antibody. The reference interval for adults 20 years and older is 0.8 to 1.7 ng/dL (10.3-21.9 pmol/L) (23).

#### Thyroid-stimulating hormone

TSH was measured using the Abbott Architect TSH assay (Catalog # 7K62, RRID:AB_2883972, Abbott Diagnostics, Chicago, IL) according to the manufacturer's instructions (24). The assay is a 2-step sandwich chemiluminescent immunoassay in which TSH in the patient sample is sandwiched between a capture antibody and acridinium-labeled antibody specific for TSH. The reference interval for adults 20 years and older is 0.35 to 4.94 mIU/L (24).

## Results

### TRAb/TSI-BA Order Patterns

**Figure 3.**
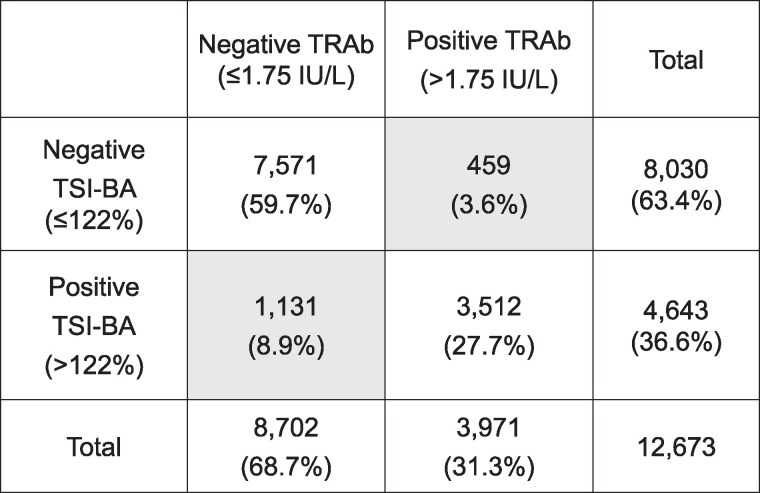
Number of samples (%) from individual patients with paired orders for TRAb and TSI-BA tests, by result. Discordant results are shaded gray.

To understand the prevalence of paired orders, all orders for TRAb or TSI-BA within the determined timeframe were reviewed. A total of 28 305 orders for TRAb only and 47 794 orders for TSI-BA only were received: 12 673 (14% of the individual encounters, n = 88 772) had orders for both TRAb and the TSI-BA. In all 12 673 samples measured by the Roche TRAb assay and TSI-BA, the Spearman correlation, r_s_, showed strong positive correlation between the 2 methods (r_s_ = 0.76, *P* < .0001). When TRAb/TSI-BA were ordered on the same encounter, qualitative agreement between tests was 87.5%. Of the discordant results for TRAb/TSI-BA, 1131 (8.9%) were negative for TRAb but positive by TSI-BA, while 459 (3.6%) were positive for TRAb but negative by TSI-BA (Fig. 3).

Using 3 times the cutoff for test negativity substantially increased agreement between the 2 assays (Table S1 (25)). Of 1590 total discordant results observed, 86% were a low positive result on 1 test, while negative by the other. Excluding those discordances due to low positive results near the cutoff (<3 times the ULN), 98.3% agreement was observed between TRAb/TSI-BA. The remaining discordances included 82 (0.6%) samples with a high positive TSI-BA but negative TRAb result and 140 (1.1%) samples with a high positive TRAb but negative TSI-BA result. Thus, most qualitative discordances between TRAb/TSI-BA were due to results near the established clinical cutoff.

### Clinical Correlation of Discordant Patient Results in TRAb/TSI-BA Orders

To determine if 1 test performed better in the discordant cases, we performed a chart review to evaluate the patient's FT4 and TSH (if available) concentration at the time of antibody testing, thyroid-related clinical diagnosis, and thyroid medication prescribed at time of testing (Table 1). Of 12 673 paired TRAb/TSI-BA orders, 152 encounters (representing 151 unique patients) had FT4 testing performed during the same encounter. These patient results were further investigated for qualitative agreement between the TRAb/TSI-BA result. Overall, 20 (13.2%) results were discordant at manufacturer-defined cutoffs; 12 (7.9%) were negative by TRAb but positive by TSI-BA, and 8 (5.3%) were positive by TRAb but negative by TSI-BA.

**Table 1.** TRAb/TSI-BA discordant patient result summary

Patient	TRAb (IU/L)	TSI-BA (%)	TSH (mU/L)	FT4 ng/dL (pmol/L)	Clinical diagnosis	Thyroid medication prescribed
1	**1.76**	93	0.86	1.2 (15.5)	None	None
2	**2**.**58**	94	0.60	1.3 (16.8)	Hyperthyroid	Methimazole
3	**3**.**11**	97	0.69	1.4 (18.1)	Transient hyperthyroid with coinciding illness	None
4	**2**.**67**	103	0.52	1.4 (18.1)	Transient hyperthyroid	None
5	**5**.**09**	116	**0**.**30**	1.2 (15.5)	Subclinical hyperthyroid	None
6	**1**.**94**	106	**0**.**33**	1.5 (19.4)	Thyroid dysfunction	Levothyroxine
7	**3**.**26**	119	**<0**.**01**	**1.8** (**23.2)**	Graves disease	None
8	**6**.**09**	106	**<0**.**01**	**2.4** (**31.0)**	Graves disease	None
9	1.41	**349**	NA	0.4 (5.2)	Graves disease	Methimazole
10	<0.9	**143**	0.71	1.0 (12.9)	Thyroiditis	None
11	1.67	**146**	2.41	1.2 (15.5)	Hyperthyroid	Methimazole
12	<0.9	**130**	0.87	1.3 (16.8)	Graves disease	Methimazole
13	<0.9	**129**	0.80	1.4 (18.1)	Graves disease	Methimazole
14	<0.9	**124**	2.84*^a^*	2.4*^a^* (31.0)	Newborn to mom with Graves	None
15	1.03	**157**	**<0**.**01**	1.6 (20.6)	Hyperthyroid	None
16	0.97	**158**	**<0**.**01**	1.7 (21.9)	Graves disease	None
17	1.23	**159**	**<0**.**01**	**2.3** (**29.7)**	Graves disease	None
18	0.96	**291**	**<0**.**01**	**2.6** (**33.5)**	Graves disease	None
19	<0.9	**191**	**<0**.**01**	**3.5** (**45.2)**	Hyperthyroid	None
20	<0.9	**>500**	**<0**.**01**	**4.8** (**61.9)**	Graves disease	None

Results outside of assay-specific reference intervals are noted in bold.

Abbreviations: FT4, free thyroxine; TRAb, thyroid-stimulating hormone receptor antibody; TSH, thyroid-stimulating hormone; TSI-BA, thyroid-stimulating immunoglobulin bioassay.

^*a*^Reference intervals for this patient (pediatric) were TSH 0.88-5.42 mU/L and FT4 0.7-2.7 ng/dL (9.0-34.8 pmol/L).

Two of the 8 patients with a positive TRAb but negative TSI-BA had elevated FT4 and suppressed TSH. These patients were also the only 2 confirmed GD diagnoses with a negative TSI-BA result. Two additional patients had normal FT4, but TSH just below the lower reference limit, consistent with preclinical hyperthyroidism; however, 1 of those patients was being treated with levothyroxine for unspecified thyroid dysfunction. Lastly, 2 other TRAb positive and TSI-BA negative patients had transient hyperthyroidism that fully resolved.

Conversely, the clinical correlation in the TRAb negative, TSI-BA positive group was more consistent with a history of GD and hyperthyroidism. In that group, 7 of 12 patients had a GD diagnosis, 3 had unspecified hyperthyroidism, 1 case of thyroiditis, and 1 newborn born to a mother with GD (likely placental transfer). Four of these individuals had elevated FT4 and 6 had suppressed TSH. Four of the 8 patients with normal FT4 were being treated with methimazole (MMI), an antithyroid medication, which may have contributed to the normal FT4 and TSH in these patients.

### TRAb/TSI-Br Order Patterns

**Figure 4.**
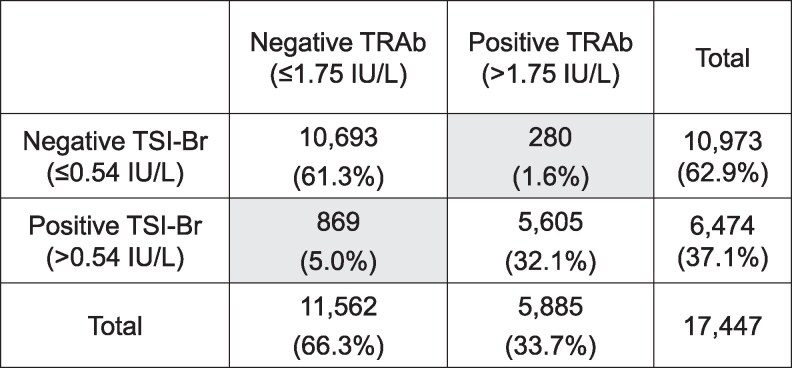
Number of samples (%) from individual patients with paired orders for TRAb and TSI-Br tests, by result. Discordant results are shaded gray.

To understand the prevalence of paired orders, all orders for TRAb or TSI-Br within the determined time were reviewed: 29 985 orders for TRAb only and 52 891 orders for TSI-Br only were received. During that time, 17 447 (17% of the individual encounters, n = 100 323) orders for both TRAb/TSI-Br were received. In all 17 447 samples measured by the Roche TRAb assay and Siemens Immulite TSI-Br, the Spearman correlation, showed a very strong positive correlation between the 2 methods (r_s_ = 0.87, *P* < .0001). The Bland–Altman analysis showed acceptable agreement, with a mean bias of 0.71 IU/L and confidence limits of −7.46 and 8.88 IU/L. The qualitative agreement between TRAb/TSI-Br was better than that of TRAb and the TSI-BA at 93.4%. Of the 17 447 paired orders, there were 1149 (6.6%) discordant results, where 869 (5.0%) were negative for TRAb but positive by TSI-Br and 280 (1.6%) were positive for TRAb but negative by TSI-Br (Fig. 4).

Using 3 times the cutoff for test negativity resulted in a higher concordance rate of 98.9% (Table S2 (25)). Of the 1149 discordant results, 84% were a low positive result on 1 test, while negative by the other, suggesting some variability between assays closer to the cutoffs. Excluding discordances due to low positive results near the cutoff (<3 times the ULN), the remaining discordances included 150 (0.9%) samples with a high positive TSI-Br but negative TRAb result, and 29 (0.2%) samples with a high positive TRAb but negative TSI-Br result, showing increased agreement between results further from the established cutoffs (Table 2 (25)).

### Clinical Correlation of Discordant Patient Results for TRAb/TSI-Br Orders

To determine if 1 test correlated better with clinical data in discordant cases, we performed chart review to evaluate FT4 and TSH concentrations at the time of antibody testing, thyroid-related clinical diagnosis, and thyroid medication prescribed at time of testing (Table 2). Of the 17 447 paired orders, 220 encounters (152 unique patients) also had FT4 and TSH testing performed during the same encounter. These encounters were further investigated for qualitative agreement between TRAb/TSI-Br results at manufacturer-defined cutoffs. Sixteen (7.3%) encounters representing 15 different patients were discordant; 12 (11 patients) (5.5%) were negative by TRAb but positive by TSI-Br, while 4 (1.8%) were positive by TRAb and negative by TSI-Br.

**Table 2.** TRAb/TSI-Br discordant patient result summary

Patient	TRAb (IU/L)	TSI-Br (IU/L)	TSH (mU/L)	FT4 ng/dL (pmol/L)	Clinical diagnosis	Thyroid medication prescribed
1	**2.91**	<0.10	0.51	1.4 (18.1)	Subclinical hyperthyroid	None
2	**1**.**87**	<0.10	**0**.**02**	1.6 (20.6)	Subclinical hyperthyroid	None
3	**1**.**78**	0.26	**0**.**03**	1.4 (18.1)	Thyroiditis	None
4	**5**.**23**	0.33	**0**.**02**	1.6 (20.6)	Fluctuating hypo-/hyperthyroid	None
5	<0.9	**0**.**56**	2.19	1.5 (19.4)	Graves disease	Methimazole
6	1.21	**0**.**99**	4.08	1.2 (15.5)	Graves disease	Methimazole
7	1.72	**1**.**32**	2.17	0.9 (11.6)	Graves disease	Methimazole
8	1.7	**0**.**57**	**<0**.**01**	1.2 (15.5)	Subclinical hyperthyroidism	None
9	1.23	**0**.**75**	**<0**.**01**	1.4 (18.1)	Graves disease	None
10	1.65	**0**.**82**	**<0**.**01**	1.3 (16.8)	Hyperthyroidism (unspecified etiology)	None
11	1.24	**1**.**13**	**<0**.**01**	1.7 (21.9)	Hyperthyroidism	Methimazole
12	<0.9	**0**.**59**	1.79	**1.8** (**23.2)**	Graves disease	None (Methimazole in past)
13	<0.9	**1**.**23**	**<0**.**01**	**3.5** (**45.2)**	Hyperthyroidism	Methimazole
14	1.66	**0**.**98**	**<0**.**01**	**3.0** (**38.7)**	Graves disease	None
15	1.27	**0**.**63**	**<0**.**01**	**2.2** (**28.4)**	Graves disease	None

Results outside of assay-specific reference intervals are noted in bold.

Abbreviations: FT4, free thyroxine; TRAb, thyroid-stimulating hormone receptor antibody; TSH, thyroid-stimulating hormone; TSI-Br, thyroid-stimulating immunoglobulin bridge immunoassay.

In TRAb positive patients, none had elevated FT4, nor were they prescribed antithyroid medications; however, 3 of the 4 had suppressed TSH concentrations. Two of those were diagnosed as subclinical hyperthyroid, while 1 had thyroiditis, and 1 patient was fluctuating hypo- and hyperthyroid. In those that were TRAb negative, TSI-Br positive (n = 11), 7 had a GD diagnosis, while the remaining 4 had hyperthyroidism of unspecified etiology. Three individuals with GD had elevated FT4 concentrations, 2 of whom also had suppressed TSH. All patients with GD with normalized FT4 and TSH concentrations were being treated with MMI at the time of testing.

### Cost Analysis

The average list price of TRAb testing (from 4 laboratories) was $170 and the TSI-BA (from 2 laboratories) was $553 (Table 3). The total cost was $723 when both tests were ordered. We observed 12 673 paired orders for TRAb/TSI-BA, equating to a total cost of $9 162 579. That cost is 325% more than the cost of testing if TRAb was ordered alone ($2 154 410) and 31% more than if TSI-BA was ordered alone ($7 008 169).

**Table 3.** Predicted cost burden due to paired ordering of TRAb and TSI during the same encounter

Tests ordered	No. of orders	Costs as ordered, $	Cost if ordered as TRAb only, $	Cost if ordered as TSI only, $	Unnecessary costs incurred, $
TRAb/TSI-BA	12 673	9 162 579	2 154 410	7 008 169	2 154 410–7 008 169
TRAb/TSI-Br	17 447	6 699 648	2 965 990	3 733 658	2 965 990–3 733 658

Abbreviations: TRAb, thyroid-stimulating hormone receptor antibody, TSI, thyroid-stimulating immunoglobulin; TSI-BA, TSI bioassay; TSI-Br, TSI bridge immunoassay.

The average list price of the TSI-Br test (from 2 laboratories) was $214; therefore, the total cost was $384 when ordered with TRAb (Table 3). In total, we observed 17 447 paired orders for TRAb/TSI-Br, which equated to $6 699 648 in testing costs. These paired orders cost 126% more than if TRAb was ordered alone ($2 965 990) and 79% more than TSI-Br alone ($3 733 658).

## Discussion

There are several ways to measure TRAbs; however, ATA guidelines for diagnosis and management of hyperthyroidism (6) do not specify what test should be used. Without an understanding of the correlation between methods, one cannot determine the clinical or economic impacts associated with ordering paired tests. In this study, we reviewed how frequently both TRAb and TSI were ordered during the same encounter, evaluated the clinical results between tests, and examined the economic impact of paired orders. We found greater than 10% of all orders for TRAb and/or TSI had both tests ordered during the same encounter. By Spearman correlation, we showed TRAb had strong correlation with TSI-BA (r_s_ = 0.76) and very strong correlation with TSI-Br (r_s_ = 0.87). Multiple previous studies evaluating the Roche TRAb assay against the TSI-Br assay in individuals with newly diagnosed GD also showed strong positive correlation between TRAb and TSI-Br (r_s_ = 0.810-0.920, *P* < 0.001) (26-29). Furthermore, we observed more than 90% qualitative agreement between TRAb/TSI in paired orders. This high rate of correlation and agreement suggests both tests provide similar clinical information.

Closer examination of discordant results showed that most differences were due to results near the manufacturer defined cutoffs for each assay. Specifically, we observed more low positive results by TSI that were negative by TRAb. This agreed with previous reports showing slightly higher positivity in TSI when compared head to head with TRAb tests, which may be attributed to higher clinical sensitivity of the TSI assays investigated (16, 17, 28, 30-32). In addition to differences in clinical sensitivity between assays, different assay specificities, TSHR epitope targeted, and elements unique to individual patients (such as treatment status/response) are likely contributing to the small percentage of discordance observed.

Clinically, when TSIs are present, one would expect overstimulation of the TSHR and excess production of the thyroid hormones, T4 and triiodothyronine, provided the thyroid can respond (iodine sufficient and not substantially damaged or removed). The pituitary is then signaled via a negative feedback loop to decrease the production of TSH in response to thyroid hormone elevations. In the laboratory, this may be recognized by FT4 concentrations above the upper reference limit and TSH values below the lower reference limit (33). Patients with previously diagnosed GD, however, may be prescribed antithyroid medication(s) (such as MMI) to inhibit the synthesis of thyroid hormone and normalize FT4 and TSH concentrations (34). Indeed, this was observed in our chart review of discordant results. Taking both datasets together, there were 7 times more patients with a documented GD diagnosis in the TSI-positive group (n = 14) compared to the TRAb-positive group (n = 2). Fourteen TSI-positive (BA or Br) patients had elevated FT4 and/or suppressed TSH compared to 7 TRAb-positive patients. Furthermore, all TSI-positive GD patients with FT4 and TSH concentrations within the reference interval were prescribed MMI at the time of testing, suggesting medication-mediated control of the thyroid. This finding is consistent with previous work that found in cases with inconsistent results, the Siemens TSI-Br result was a more accurate representation of the patients clinical status than the Roche TRAb result in patients being treated for GD (16, 32). In contrast, the TRAb-positive group was dominated by patients with subclinical or transient hyperthyroidism, only 2 of whom were prescribed thyroid-specific medication. One of those was being treated with levothyroxine for unspecified thyroid dysfunction. In this case, the patient was likely hypothyroid when prescribed the levothyroxine, which may be consistent with the presence of blocking TRAb that are detected in the TRAb assay, but not in the TSI-BA. Taken together, these data suggest the TSI assays may correlate better with clinical history and other biomarkers of GD than the TRAb assay, which is in agreement with prior studies (12, 15, 16, 28). In contrast, TRAb may also be positive in other thyroid conditions such as hypothyroidism, as would be expected in the presence of blocking antibodies, or transient hyperthyroidism.

Given these data, we propose that ordering TRAb and TSI on the same encounter is likely redundant and an excessive use of health care dollars in most cases. This study found that the authors’ laboratory received paired orders for TRAb/TSI in 14% to 17% of all orders received for TRAb or TSI. Based on the average list price and the redundant order frequency, a 31% to 325% increase in laboratory testing costs would be incurred by ordering both TRAb and TSI, equaling over $2 to 7 million dollars in potentially unnecessary health care dollars in the dataset we evaluated, which represents only a portion of the annual thyroid antibody testing performed in the United States. This cost analysis was based on list price for the assays, with the caveat that contracted pricing will routinely be lower, but it still provides an approximation of the maximum cost associated with redundant ordering. National health care expenditure in the United States grew to $4.5 trillion in 2022 and is projected to continue increasing faster than that of the gross domestic product (35, 36). With climbing health care costs and estimates of wasteful spending ranging from $600 billion to over $1.9 trillion per year (37), there is increasing need to reduce unnecessary expenditure of health care dollars. This study highlights how improving test utilization has the potential to significantly reduce wasteful spending with millions of dollars saved by altering order patterns for just 1 test at a single reference laboratory.

The key strengths of this study were that we (1) evaluated a large dataset of more than 30 000 paired orders for TRAb/TSI, (2) included chart review of discrepant results that allowed for clinical correlation of test results with thyroid status, diagnoses, and medications, and (3) compared TRAb results to 2 different TSI assay formats available to providers. Despite its strengths, our study does have a few limitations. One limitation to our study was the lack of information describing why paired orders were placed for the patients included in our analysis. A survey or interview of users may explain ordering patterns and how test results were interpreted. Additionally, a prospective review of the patient's clinical status and treatment postantibody testing may provide insight into the concordance of TRAb vs TSI over the course of disease. Secondly, our dataset for clinical correlation with antibody testing results was small and limited to findings documented in the medical chart. This included physician and nurse visit notes, documented diagnoses, and laboratory testing. Clinical data or diagnoses not recorded in the medical record would be missed in our analysis. Future studies expanding the investigation of the clinical correlation with TRAb/TSI test results in a larger cohort may be insightful.

In summary, this study highlighted redundant ordering of TRAb and TSI testing in approximately 15% of all encounters for TSHR autoantibody testing in the timeframe examined. Qualitative assessment of paired orders showed good overall agreement, suggesting that ordering both tests for an individual patient during the same encounter may not provide clinical or analytical benefit and was associated with substantially higher health care costs. In the rare instances when discordant results were observed, TSI assays correlated better with clinical history and other biomarkers of GD than the TRAb assay. The evidence presented here may provide useful ordering guidance to clinicians and inform future clinical practice guidelines.

## Data Availability

Restrictions apply to the availability of the data generated or analyzed during this study to preserve patient confidentiality. The corresponding author will on request detail the restrictions and any conditions under which access to some data may be provided.

## Abbreviations

cAMP: cyclic adenosine monophosphate
FT4: free thyroxine
GD: Graves disease
MMI: methimazole
TSHR: thyroid-stimulating hormone receptor
TRAb: TSHR antibody
TSI: thyroid-stimulating immunoglobulin
TSI-BA: TSI bioassay
TSI-Br: TSI bridge immunoassay
ULN: upper limit of normal
